# Effects of elevated CO_2_ on the fitness and potential population damage of *Helicoverpa armigera* based on two-sex life table

**DOI:** 10.1038/s41598-017-01257-7

**Published:** 2017-04-25

**Authors:** Jinping Liu, Wenkun Huang, Hsin Chi, Chonghui Wang, Hongxia Hua, Gang Wu

**Affiliations:** 1 Hubei Key Laboratory of Insect Resource Utilization and Sustainable Pest Management, College of Plant Science and Technology, Huazhong Agricultural University, Wuhan, 430070 China; 2 State Key Laboratory for the Biology of Plant Diseases and Insect Pests, Institute of Plant Protection, Chinese Academy of Agricultural Sciences, Beijing, 100193 China; 3 Department of Plant Production and Technologies, Faculty of Agricultural Sciences and Technologies, Ömer Halisdemir University, Niğde, Turkey

## Abstract

We evaluated the direct effects of three different atmospheric CO_2_ concentrations (380 ppm, 550 ppm and 750 ppm) on the population parameters of the cotton bollworm, *Helicoverpa armigera* fed an artificial diet. The life history and fitness of *H*. *armigera* were analyzed using an age-stage, two-sex life table. Our results showed significantly longer larval durations and lower female pupal weight under elevated CO_2_ than under ambient CO_2_. Additionally, the fecundity of *H*. *armigera* was lower under elevated CO_2_ than under ambient CO_2_. *H*. *armigera* reared under elevated CO_2_ conditions showed lower intrinsic and finite rates of increase but higher net consumption and finite consumption rates than *H*. *armigera* reared under ambient CO_2_ conditions. According to population projections, a much smaller total population size and reduced consumption capacities would be expected in an elevated CO_2_ atmosphere due to higher mortality and lower growth rate compared with ambient CO_2_ levels. These results indicate that the fitness of and potential damage caused by *H*. *armigera* would be affected by increased CO_2_ relative to ambient CO_2_ concentrations. Additional studies on the long-term direct and indirect effects of elevated CO_2_ levels on *H*. *armigera* are still needed.

## Introduction

*Helicoverpa armigera* (Hübner; Lepidoptera: Noctuidae) is an extremely destructive and economically important pest of diverse agricultural commodities throughout the world. To date, it has been recorded on more than 68 host plant families^1, 2^. The larvae prefer to feed on younger leaves as well as flower structures, while female adults prefer to lay their eggs on whichever plants happen to be the locally abundant host species^3^. Depending on which species is selected, this behavior may cause a sizable economic loss.

Ambient carbon dioxide concentrations had increased to as high as 380 ppm by 2005 and have been predicted to reach at least 550 ppm by the year 2050 and to double by the end of the 21^st^ century due to continuing high levels of fossil fuel consumption and various agricultural practices^4^. This increase may directly affect plants by altering the chemical composition of the air, resulting in modifications to plant secondary metabolism^5^. Increases in the C/N ratio have been found in plants growing under elevated CO_2_, which are expected to affect C-based secondary chemistry. These changes also reduce the nutritional quality of plant tissue, resulting in decreased nitrogen concentration and increases in phenolics^6^. Due to cascade effects, such variation in host plants may indirectly and directly affect herbivores performance and population dynamics^7^. Most previously published studies have focused on the indirect effects of elevated CO_2_ on quantifiable aspects of an herbivore’s performance. These studies generally involve measurements of development, reproduction, and consumption, and they often addresses the effects of elevated CO_2_ on herbivores by altering a plant’s primary and secondary metabolism^8^. CO_2_ enrichment may also affect herbivore consumption by altering plant hormones^9, 10^. Although herbivore responses to increased CO_2_ have been relatively well-studied given the extensive interpretations reported, they are highly variable and the underlying mechanisms need to be explored further^10^. Because information regarding the direct effects of elevated CO_2_ on herbivory performance is currently lacking, additional data are needed to comprehensively understanding the influence of increased levels of CO_2_ on herbivores.

Data obtained from life table compilations provide researchers with a comprehensive understanding of the development, survivorship and fecundity of a population cohort. This types of analysis reveals the fitness of a population under variable biotic and abiotic conditions and has increasingly been used as an invaluable tool in successful biological control programs^11^. Age-stage, the two-sex life tables are currently used by many researchers in place of the traditional female-based, age-specific life table, primarily because of the ability to incorporate the male component of a population as well as the stage differentiation of individuals in the population^12^. Female-only life tables by definition ignore males, which normally account for 50% of a population and contribute significantly to its ecology. The raw data obtained from an age-stage, two-sex life table are much more credible and meaningful than those obtained from traditional age-specific life tables. Because development rates vary widely in a population, stage differentiation is critical to understanding the population ecology of insect herbivores^13^.

In this study, we investigated the fitness of and potential damage caused by a population of *H*. *armigera* reared on an artificial diet under three different CO_2_ concentrations: 380 ppm, 550 ppm, and 750 ppm. An age-stage, two-sex life table was used to analyze all historical data, including growth rate, reproduction and consumption data. The objectives of the study were to (1) determine the population parameters and consumption rate of *H*. *armigera* in response to elevated levels of CO_2_, and compare our life table data with previous life table studies^14–16^; and (2) predict the fitness of and potential damage caused by *H*. *armigera* populations resulting from anticipated future increases in CO_2_ concentrations.

## Results

### Life table of *H*. *armigera*

Out of 150 eggs reared on three CO_2_ treatments, there are 134, 133, and 127 eggs incubated in each of the 380 ppm, 550 ppm, and 750 ppm CO_2_ chambers successfully hatched within 3 days, indicating that CO_2_ enrichment did not adversely impact the egg stage of *H*. *armigera*. The total number of eggs (including hatched eggs plus un-hatched eggs) and the number of hatched eggs per individual female were recorded daily. The results showed that the total mean numbers of eggs produced in the three different treatments were 728.3, 685.8 and 591.1 for CO_2_ concentrations of 380 ppm, 550 ppm, and 750 ppm, respectively (Table 1). The mean number of hatched eggs was highest (392.5 offspring) under 380 ppm CO_2_, which was significantly greater than that in the 750 ppm treatment (228.1 offspring; Table 1). No significant differences were observed in the pre-oviposition periods (APOP) of female adults reared under the three CO_2_ concentrations, although the longest total pre-oviposition period (TPOP, which includes the pre-adult period) of *H*. *armigera* was observed in the 750 ppm treatment group (27 d).

**Table 1:** Development time, survival rate, APOP, TPOP, fecundity and pupal weight (mean** ± **SE) of artificial diet-fed *H*. a*rmiger*a in response to different CO_2_ treatments.

Measured parameter	CO_2_ concentration		
	380 ppm	550 ppm	750 ppm
Egg duration (d)	3.00 ± 0a (134)	3.00 ± 0a (133)	3.00 ± 0a (127)
L1–L4 (d)	7.18 ± 0.03a (134)	7.62 ± 0.06b (130)	7.61 ± 0.06b (116)
L5–L6 (d)	4.05 ± 0.06a (127)	4.74 ± 0.09b (110)	5.68 ± 0.13c (101)
Female pupal duration (d)	9.35 ± 0.08ab (55)	9.13 ± 0.08a (38)	9.53 ± 0.11b (34)
Male pupal duration (d)	9.80 ± 0.11a (49)	9.69 ± 0.10a (45)	10.45 ± 0.16b (40)
Female adult duration (d)	9.13 ± 0.48b (55)	7.92 ± 0.64ab (48)	6.65 ± 0.51a (34)
Male adult duration (d)	8.61 ± 0.51a (49)	7.78 ± 0.52a (45)	7.60 ± 0.42a (40)
Preadult survival rate	0.776 ± 0.036b (134)	0.624 ± 0.042a (133)	0.583 ± 0.044a (127)
APOP (d)	2.26 ± 0.21a (134)	2.17 ± 0.21a (133)	2.12 ± 0.24a (127)
TPOP (d)	25.88 ± 0.23a (43)	26.31 ± 0.25a (29)	27.00 ± 0.24b (24)
Fecundity (hatched eggs)	392.5 ± 56.6b (55)	264.1 ± 39.7ab (38)	228.1 ± 46.9a (34)
Fecundity (total eggs)	728.3 ± 76.1a (55)	685.8 ± 90.0a (38)	591.1 ± 82.7a (34)
Oviposition duration (d)	4.09 ± 0.35a (55)	4.34 ± 0.38a (38)	3.46 ± 0.27a (34)
Pupal weight (♀)/g	0.311 ± 0.005b (55)	0.291 ± 0.005a (38)	0.285 ± 0.006a (34)
Pupal weight (♂)/g	0.315 ± 0.004b (49)	0.305 ± 0.004b (45)	0.288 ± 0.007a (40)

Note: Standard errors were analyzed using 100,000 bootstraps replicates. Means followed by different letters in the same row are significantly different between different CO_2_ levels by the paired bootstrap test. APOP: the pre-oviposition period based on the female adult stage. TPOP: the total preadult stage before oviposition counted from birth.

**Figure 1:**
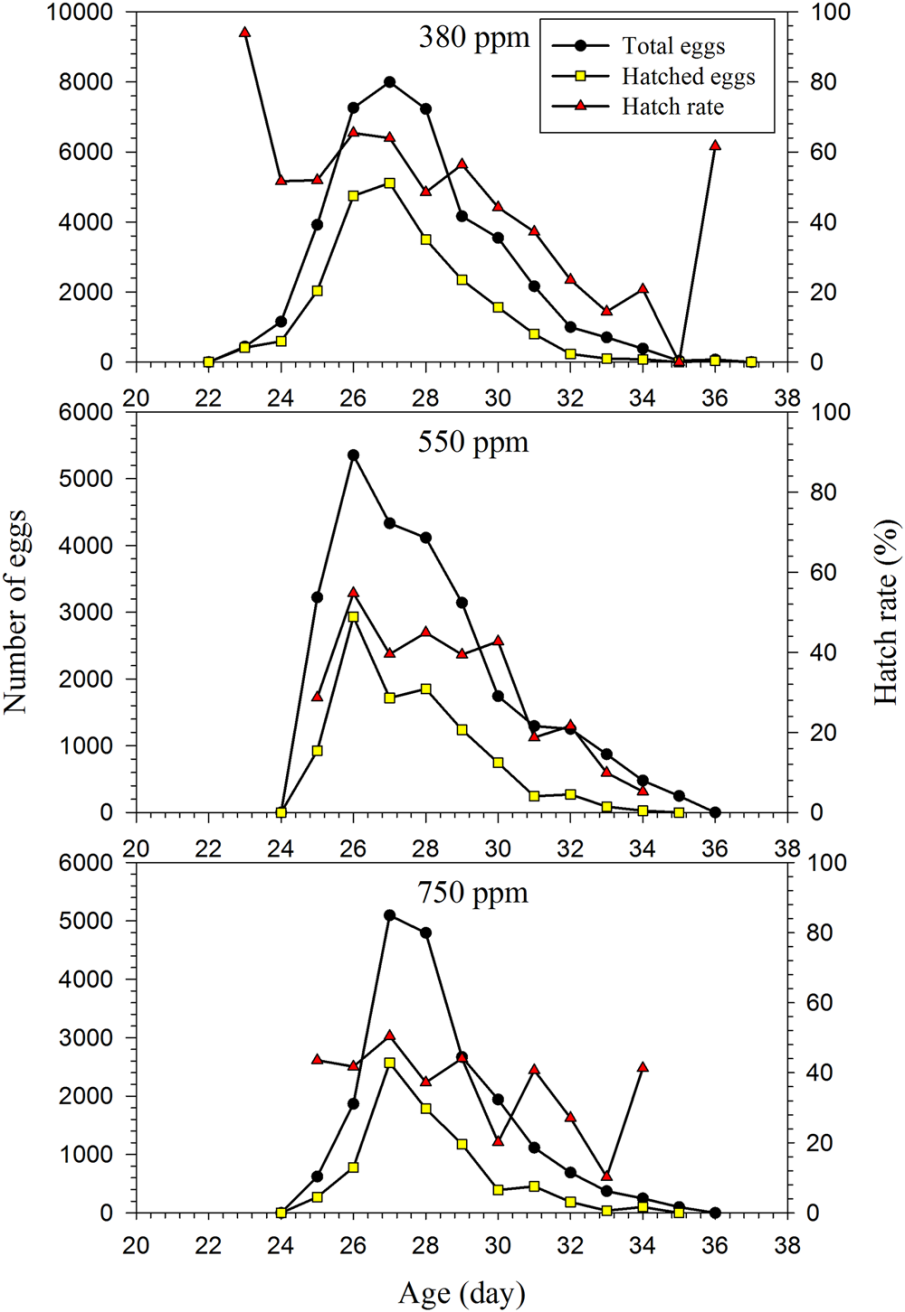
The total egg numbers and hatching rates of artificial diet-fed *H*. *armigera* on in response to different CO_2_ treatments.

**Figure 2:**
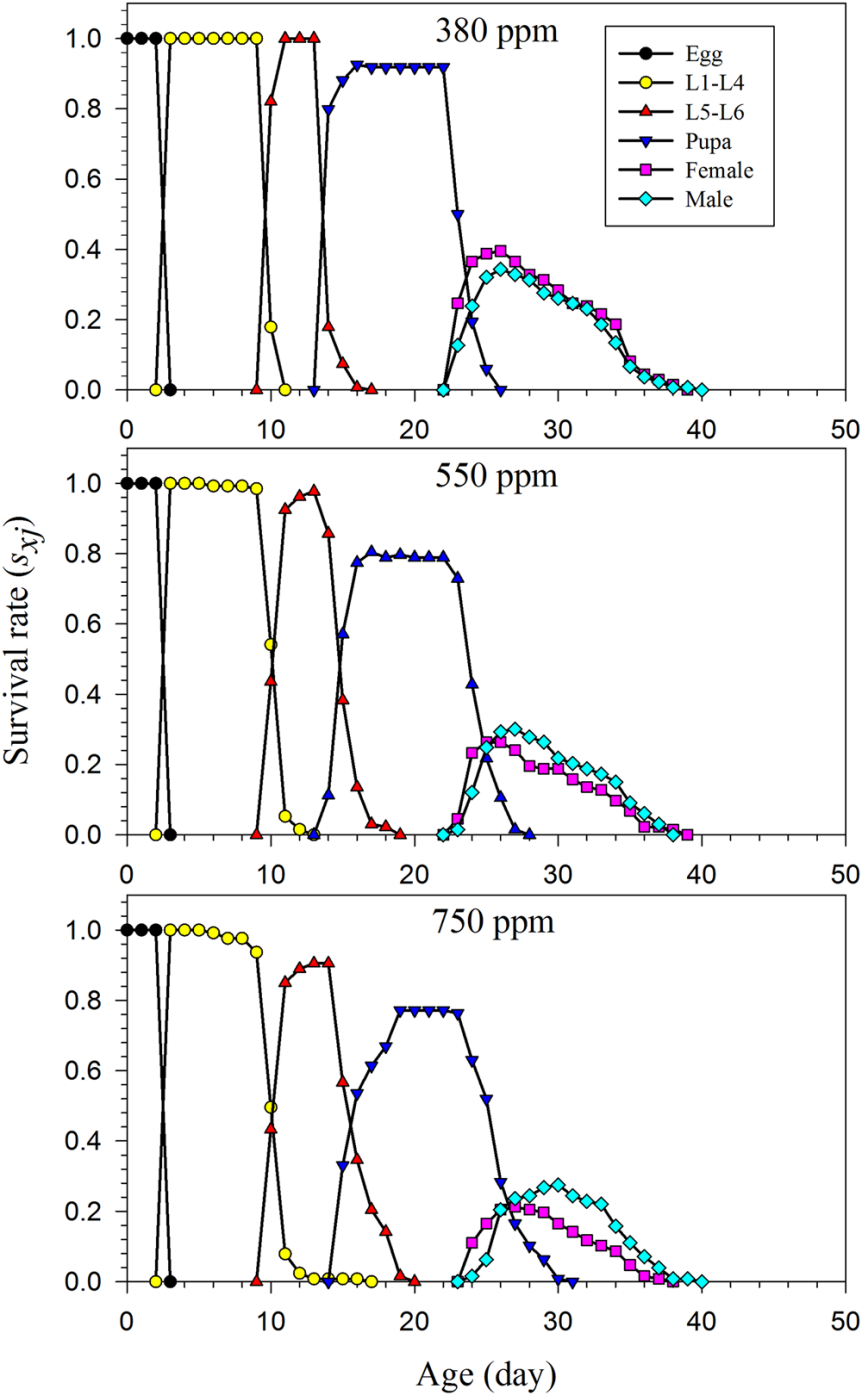
The age-stage-specific survival rate (*S*_*xj*_) of artificial diet-fed *H*. *armigera* in response to different CO_2_ treatments.

**Figure 3:**
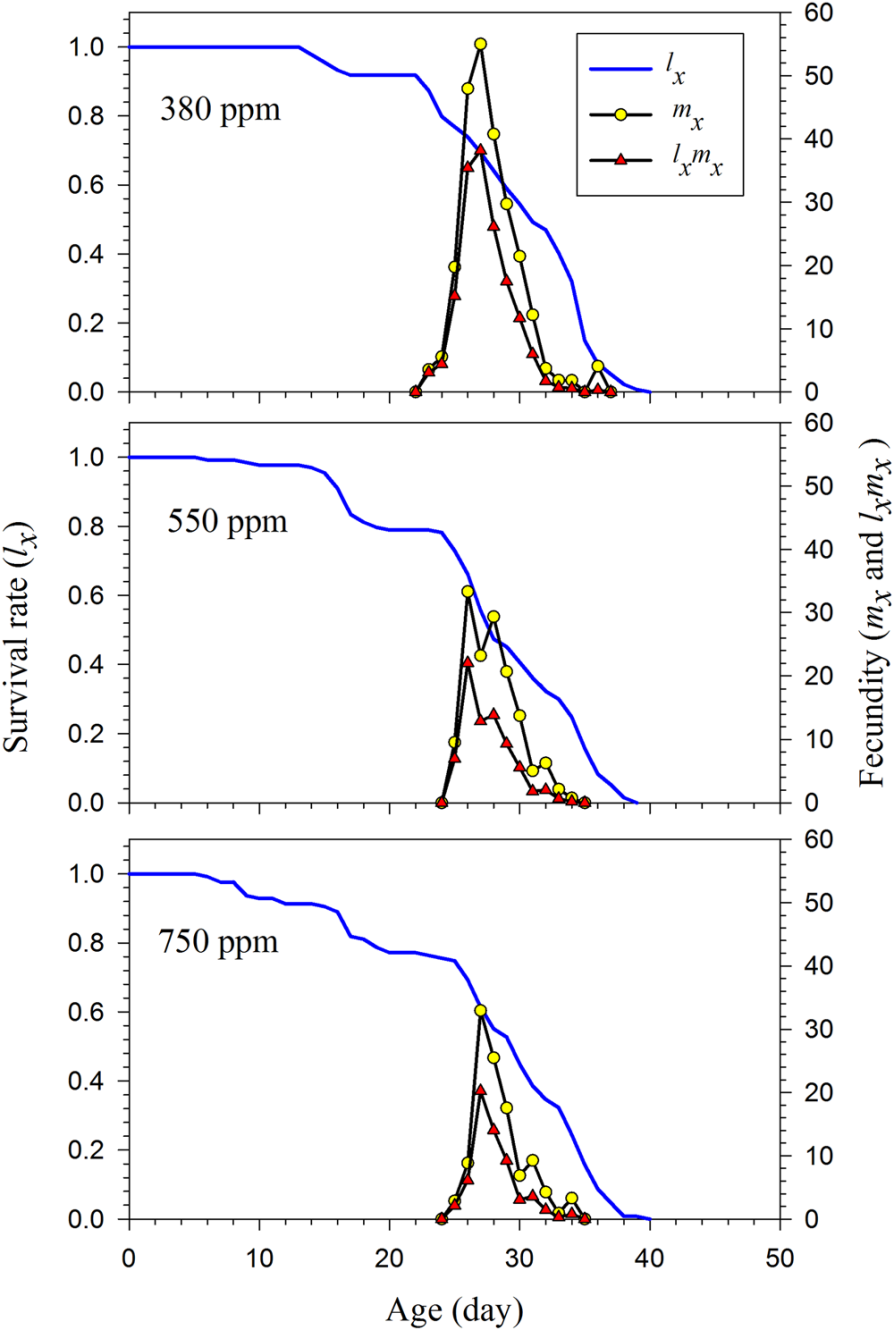
The age-specific survival rate (*l*_*x*_) and fecundity (*m*_*x*_) of artificial diet-fed *H*. *armigera* in response to different CO_2_ treatments.

**Figure 4:**
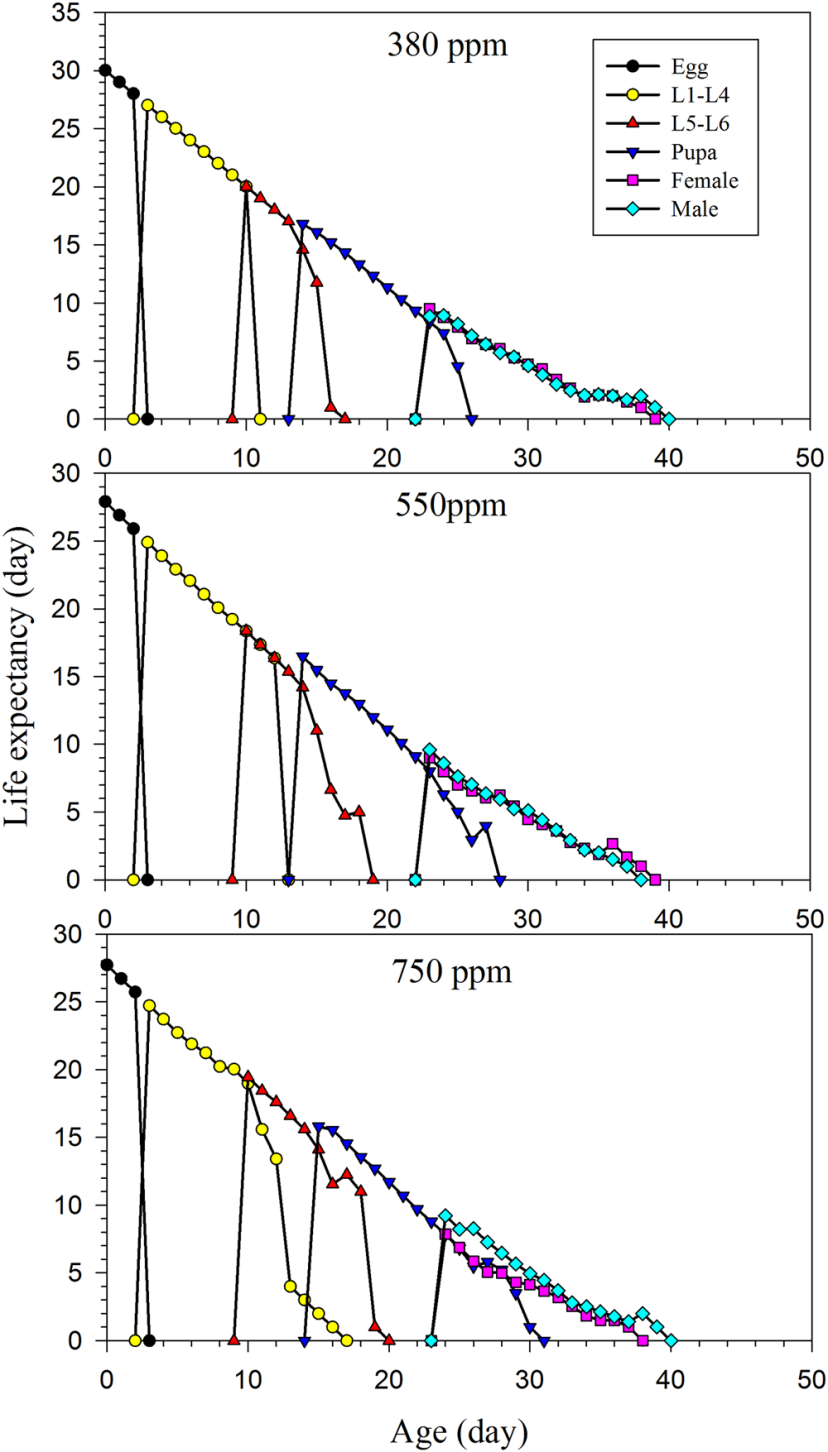
The age-stage life expectancy of artificial diet-fed *H*. *armigera* in response to different CO_2_ treatments.

The highest hatching percentage (93.9%) of females reared under the ambient CO_2_ treatments occurred on the first day of egg laying, and on day 2, the rate was only 54.8% in both the 550 ppm and 750 ppm treatments (Fig. 1). The age and stage structure and overlapping phenomena can be observed in Fig. 2. The maximal survival rate at the pupal stage was significantly higher under the 380 ppm CO_2_ treatment than for insects reared under the higher CO_2_ treatments. However, *H*. *armigera* larvae reared in 750 ppm CO_2_ experienced high mortality during the L4–L6 stages (Fig. 2). The maximal age-specific daily fecundity (*m*_*x*_) of *H*. *armigera* females was observed in the 380 ppm treatment, in which 55.0 offspring were produced through day 27 (Fig. 3). Fewer offspring were produced in the other treatments, with only 33.3 offspring produced in the 550 ppm treatment through day 26, and 32.9 offspring in the 750 ppm treatment through day 27 d. The age-stage life expectancy (*e*_*xj*_), which is the amount of time that individuals of age *x* and stage *j* would be expected to live, varied among the different CO_2_ concentrations at age *x* and stage *j* for *H*. *armigera* (Fig. 4). The life expectancy of newly laid *H*. *armigera* eggs reared in the 380 ppm CO_2_ treatment was 30.0 d, which was slightly longer than that of eggs from the 550 ppm (27.9 d) and 750 ppm (27.7 d) treatments. The peak life expectancy of first-instar larvae was 27.0 d, 24.9 d and 24.7 d in the 380 ppm, 550 ppm, 750 ppm treatments, respectively (Fig. 4). Besides, the peak life expectancy of female adult under the 550 ppm and 750 ppm treatment was higher than the male adult.

### Consumption rate of *H*. *armigera*

**Figure 5:**
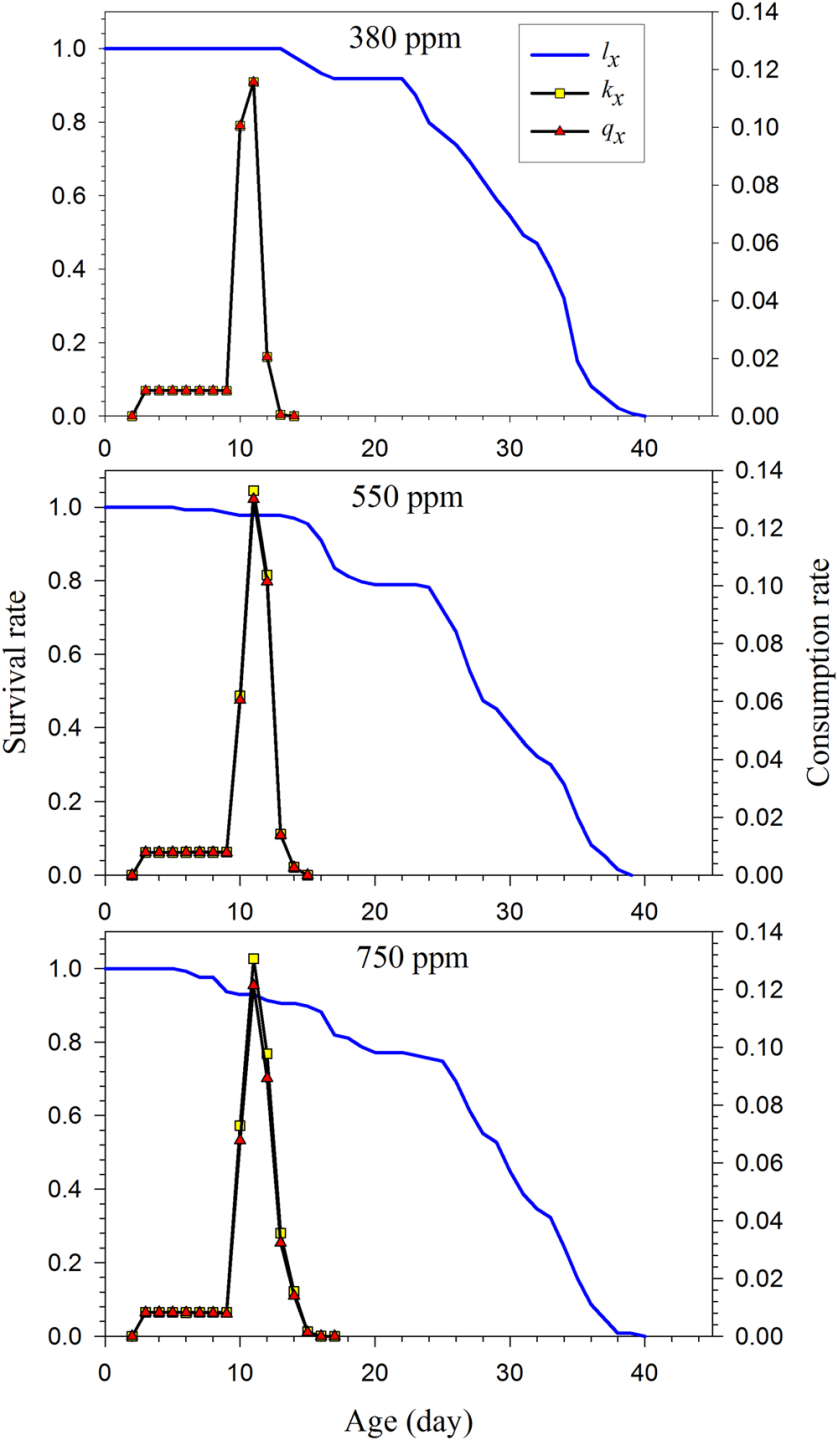
The age-specific survival rate (*l*_*x*_), age-specific consumption rate (*k*_*x*_) and the age-specific net consumption rate of artificial diet-fed *H*. *armigera* in response to different CO_2_ treatments.

The daily mean consumption rate of each stage of *H*. *armigera* larvae is shown in Table 2. The daily mean consumption rate during L5–L6 stage under the 550 ppm (0.0643 g/individual) were significantly higher than those under the 380 ppm (0.0576 g/individual) and 750 ppm (0.0616 g/individual). The net consumption rates (*C*_0_) for the 380 ppm, 550 ppm, and 750 ppm treatments were 0.2913 g, 0.3815 g, and 0.3736 g, respectively. The transformation rate (*Q*_*p*_) and finite consumption rate (*ω*) of *H*. *armigera* under elevated CO_2_ (550 ppm, 750 ppm) were significantly higher than those under ambient CO_2_. The age-specific consumption rates (*k*_*x*_) and the age-specific net consumption rates (*q*_*x*_) of *H*. *armigera* are shown in Fig. 5. The highest peaks for *k*_*x*_ (maximal age-specific consumption rate) were observed for the 550 ppm treatment (0.1307 g). Because only hatched eggs were used to reveal the true population characteristics and the high survival rate during larval stages, no difference between *k*_*x*_ and *q*_*x*_ (the age-specific net consumption rate) was detected under three treatments (Fig. 5).

**Table 2:** Net consumption rate, transformation rate and finite consumption rate (mean ± SE) of artificial diet-fed *H*. *armigera* in response to different CO_2_ treatments.

Consumption parameter	Different CO_2_ treatments		
	380 ppm	550 ppm	750 ppm
L1–L4 daily mean consumption rate (g/larva)	0.0089 ± 0.0004a (134)	0.0079 ± 0.0003a (130)	0.0081 ± 0.0005a (115)
L5–L6 daily mean consumption rate (g/larva)	0.0576 ± 0.0013a (127)	0.0643 ± 0.0020c (110)	0.0616 ± 0.0021b (101)
Net consumption rate (*C*_0_)	0.2913 ± 0.0050a (134)	0.3815 ± 0.0090b (133)	0.3736 ± 0.0140b (127)
Transformation rate (*Q*_*p*_)	0.0019 ± 0.0036a (134)	0.0048 ± 0.0011b (133)	0.0063 ± 0.0019b (127)
Finite consumption rate (*ω*)	0.0114 ± 0.0027a (134)	0.0134 ± 0.0004b (133)	0.0145 ± 0.0005b (127)

Note: Standard errors were analyzed using 100,000 bootstraps replicates. Means followed by different letters in the same row are significantly different between different CO_2_ levels using the paired bootstrap test.

### Population parameters of *H*. *armigera*

In this study, separate tallies for total eggs and hatched eggs were recorded daily to calculate and compare the age-stage-specific fecundity from the two life table data sets. The differences in population parameters generated for these two life table data sets are shown in Table 3. All of the population parameters (*r*, λ, *R*_0_, and *T*) in the life tables calculated using total eggs were higher than those in the other life tables. In life tables calculated from data excluding unhatched eggs in the parental cohort, the values for the intrinsic rates of increase (*r*), finite rates of increase (*λ*) and net reproductive rates (*R*_0_) were significantly higher in moths under the 380 ppm treatment (0.1816 d^−1^, 1.1999 d^−1^ and 161.2 offspring/female, respectively) than under the higher CO_2_ concentrations. The corresponding values obtained for the insects reared under the 550 ppm were 0.1531 d^−1^, 1.1654 d^−1^ and 75.4 offspring/female, respectively, while they were 0.1428 d^−1^, 1.1534 d^−1^ and 61.0 offspring/female, respectively, under the 750 ppm concentration. If the life table based on the total eggs, no significances were observed in the population parameters (*r*, λ, and *R*_0_) of *H*. *armigera* between 380 ppm and 550 ppm, and the mean generation time (*T*) of *H*. *armigera* under the elevated CO_2_ treatment significantly higher than that under the ambient CO_2_ treatment.

**Table 3:** Population parameter (mean ± SE) of artificial diet-fed *H*. *armigera* in response to different CO_2_ treatments.

Population parameters	380 ppm		550 ppm		750 ppm	
	Hatched eggs	Total eggs	Hatched eggs	Total eggs	Hatched eggs	Total eggs
Intrinsic rate of increase (*r*) (d^−1^)	0.1816 ± 0.0069b	0.2018 ± 0.0056b	0.1531 ± 0.0076a	0.1850 ± 0.0070ab	0.1428 ± 0.0095a	0.1735 ± 0.0073a
Finite rate (λ) (d^−1^)	1.1999 ± 0.0082b	1.2236 ± 0.0068b	1.1654 ± 0.0088a	1.2032 ± 0.0084ab	1.1534 ± 0.0101a	1.1895 ± 0.0087a
Net reproduction rate (*R*_0_) (offspring)	161.2 ± 28.6b	299.1 ± 44.8b	75.4 ± 15.3a	195.9 ± 33.1ab	61.0 ± 15.3a	153.6 ± 31.2a
Mean generation time (*T*) (d)	28.0 ± 0.2a	28.2 ± 0.2b	28.2 ± 0.2a	28.5 ± 0.2a	28.8 ± 0.4a	29.0 ± 0.3a

Note: Standard errors were analyzed using 100,000 bootstraps replicates. Means followed by different letters in the same row are significantly different among the different CO_2_ levels according to the paired bootstrap test.

### Population growth and consumption projections for *H*. *armigera*

**Figure 6:**
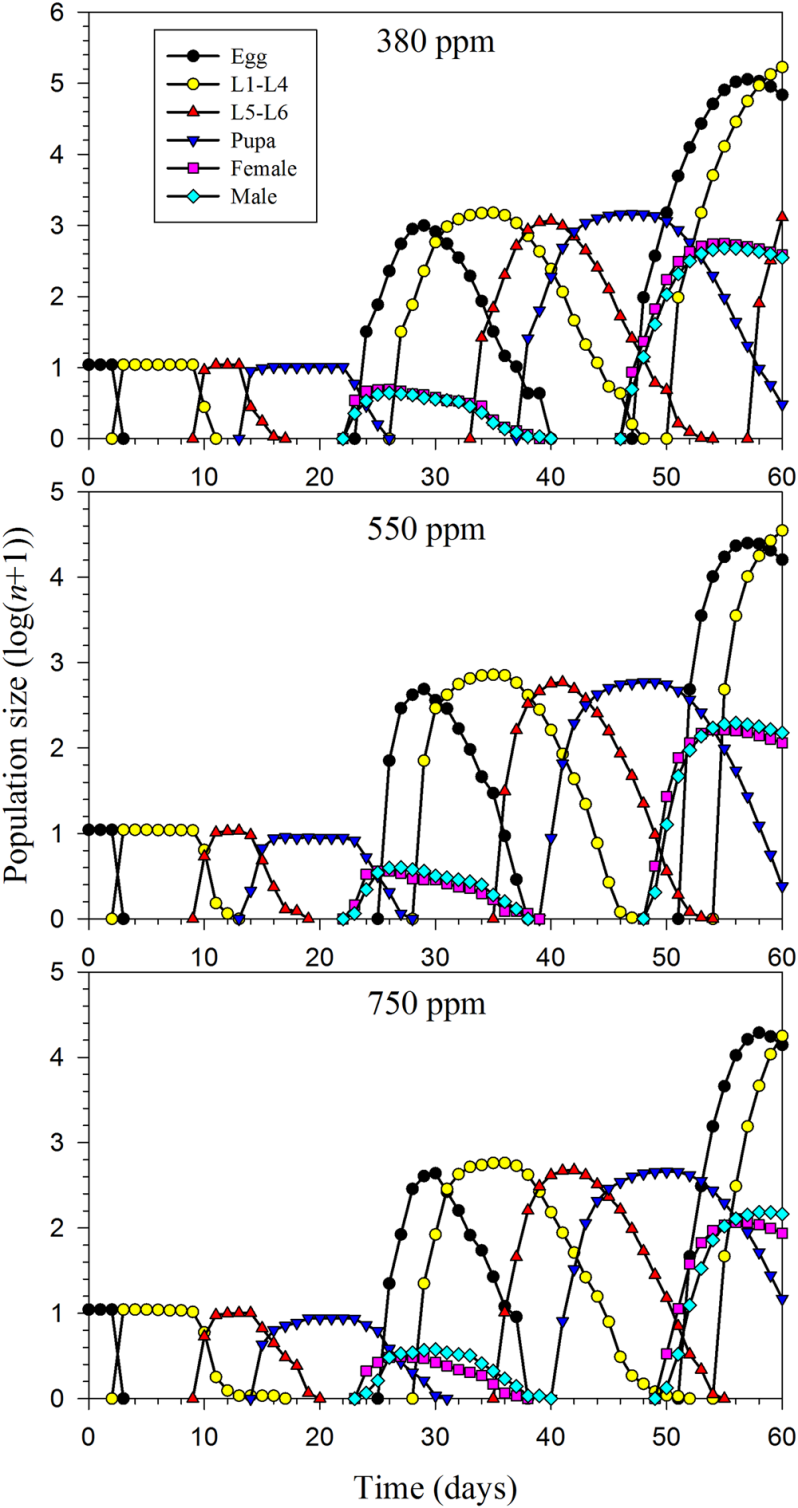
The population growth projection for *H*. *armigera* in response to different CO_2_ treatments beginning with an initial population of 10 eggs.

**Figure 7:**
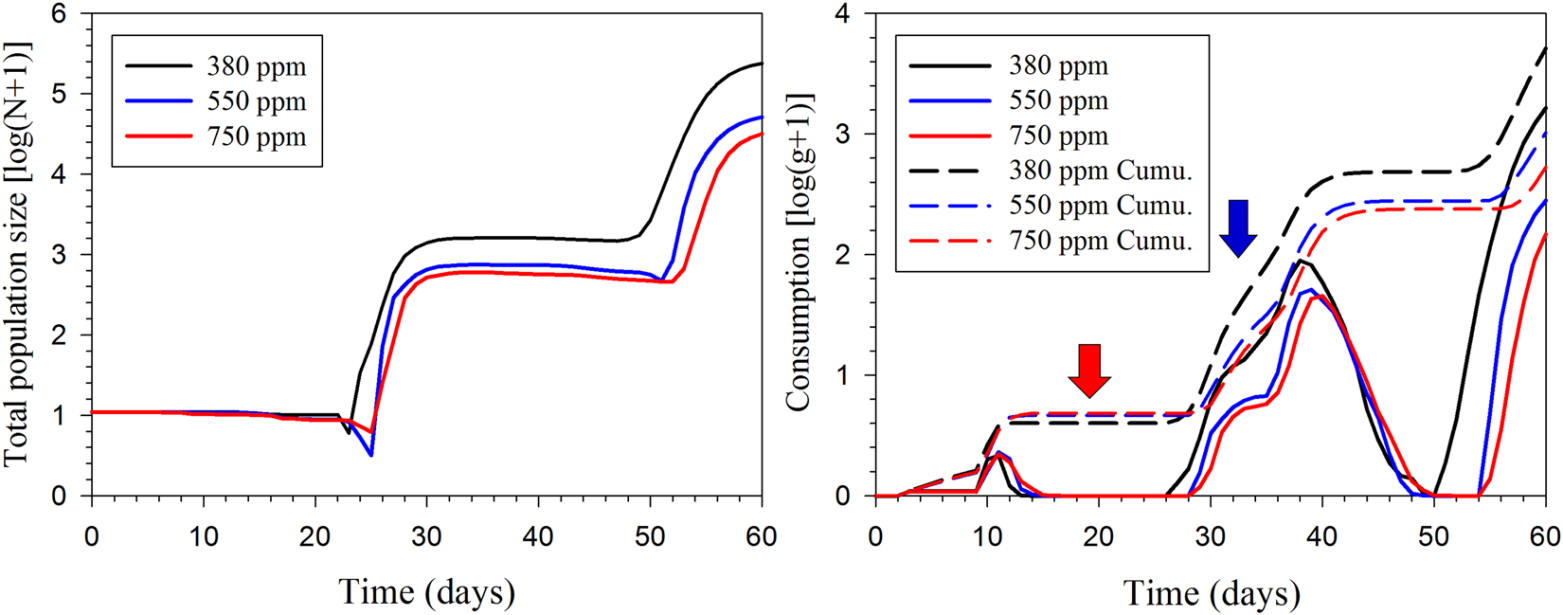
Population and consumption (daily consumption and cumulative consumption) projection of *H*. *armigera* in response to different CO_2_ treatments beginning with an initial population of 10 eggs. The red arrow shows the effect of higher net consumption rate at elevated CO_2_ conditions during the first generation. The blue arrow shows the higher consumption rate at 380 ppm CO_2_ due to the higher survival rate, rapid development, and higher intrinsic rate.

The stage structure and the both sexes are shown in the population projection based on the age-stage, two-sex life table. The projected population growths of *H*. *armigera* beginning with an initial population of 10 eggs is shown in Fig. 6. There are 1310 individuals of L4–L5 stage at time 60 d under the ambient CO_2_ treatment. However, the populations of *H*. *armigera* fed under 550 ppm and 750 ppm increased more slowly than that under the 380 ppm treatment. The total population size and consumption capacities projections of *H*. *armigera* are given in Fig. 7. The total population sizes after 60 d were projected to reach 239,527, 51,661 and 31,985 individuals under the 380 ppm, 550 ppm and 750 ppm treatments, respectively. The peak of total consumption of *H*. *armigera* in 380 ppm was high than those in the elevated CO_2_. The results showed that a much smaller total population size and consumption capacity would be expected in an elevated CO_2_ atmosphere compared with ambient CO_2_ treatment.

## Discussion

Most previously published studies focusing on the indirect effects of elevated CO_2_ on the life tables, consumption rates and population dynamics of herbivorous insects have been based on the traditional female-based, age-specific life table^17–19^. However, the response of herbivores to increased CO_2_ is still not well understood when they are reared on different host plants^20^, and additional research on the direct effects of elevated CO_2_ on herbivores should be conducted^15^. The present study measured the direct effect of elevated CO_2_ on the fitness and consumption rate of *H*. *armigera* using an age-stage, two-sex life table instead of the traditional female age-specific life table. Unlike the traditional female-based, age-specific life table, a two-sex life table can describe stage differentiation, incorporates all individuals, including males, and includes preadult mortality^12, 13^. The results demonstrated that *H*. *armigera* larvae grew at a slower rate, especially in the 5^th^ to 6^th^ instars, under elevated concentrations of CO_2_ compared with ambient CO_2_. An exception was observed for the duration of the female pupal stage, which was not affected by elevated CO_2_ levels, although significantly lower female pupal weight was found under elevated CO_2_ conditions compared with ambient CO_2_ conditions (Table 1). Because a reduction in female pupal weight has been shown to be a possible limiting factor for potential egg production in other studies^21–23^, it was not unexpected for differences in the fecundity rates to be observed in the different CO_2_ treatments.

In this study, the numbers of hatched and total eggs were counted daily, based on the observation that hatching rates vary depending on the age of female adults (Fig. 1), as some percentage of the eggs laid are produced by young unmated females or females that are past their prime fertility and consequently produce infertile eggs^24^, Chi *et al*.^25^ noted when unhatched eggs are included in the totals values employed for calculating life table parameters, the inclusion of population projections erroneously overestimates the population size, demonstrating that only the life table data based on hatched eggs will produce accurate and meaningful population parameters and reliable population projections.

Higher net consumption rates (*C*_0_) were observed under the elevated CO_2_ treatments when survival rates, longevity and consumption rates were incorporated. The transformation rates (*Q*_*p*_) for *H*. *armigera* reared under the 380 ppm, 550 ppm, and 750 ppm treatments were 0.0019, 0.0048 and 0.0063, respectively, indicating that the *H*. *armigera* populations needed to consume more to produce offspring under elevated CO_2_ conditions than under ambient CO_2_ conditions (Table 2). Chi *et al*.^26^ indicated that finite and intrinsic growth rates alone could not describe potential population damage. Therefore, they introduced the finite consumption rate (*ω*), combining the finite rate, the stable age-stage distribution, and the age-stage specific consumption rate. In the present study, the finite consumption rates in the 550 ppm and 750 ppm CO_2_ treatments were 0.0134 and 0.0145, respectively, which were significantly higher than those in the ambient CO_2_ treatment (0.0114; Table 2).

Population projections based on an age-stage, two-sex life table provide details such as fitness and potential damage that are essential for realistic population growth predictions. The stage structure is essential in projecting pest populations and in population ecology due to changing susceptibilities to various stimuli that impact different stages and ages. The male component of a population is not less important than the female portion. Because males of the majority of species consume as many resources as the female, there is no valid reason for excluding them from life table calculations. This extra information provided when an age-stage, two-sex life table is utilized produces the most economical and efficient control strategies, which can then be precisely applied at the appropriate time according to the simulation^12, 26^. In this study, despite the higher finite consumption rate observed in the elevated CO_2_ treatments, the population size and total consumption levels were lower than those under ambient CO_2_ (Figs 6 and 7). These results may be explained by the longer developmental period and higher mortality of the larvae. On the basis of these calculations, we conclude that *H*. *armigera* would exhibit lower fitness and cause less damages in the future scenarios with increasing CO_2_ concentrations.

Several previous studies have focused on the indirect effects of elevated CO_2_ on performances of *H*. *armigera* ^15, 27–29^. Chen *et al*.^27^ found that *H*. *armigera* reared on transgenic cotton grown under elevated CO_2_ exhibited higher consumption, a longer larval duration, lower fecundity, and a decreased mean relative growth rate. These observations are consistent with the results of Yin *et al*.^15^ in a study in which *H*. *armigera* was reared on maize, and both studies suggested that the damage caused by *H*. *armigera* could be serious. These findings are unlike the results of Wu *et al*.^28^, who indicated that the net damage caused by *H*. *armigera* on wheat grown under elevated CO_2_ would be lower because the increased relative consumption rate would be counteracted by delayed development and decreased fecundity. In addition, Yin *et al*.^29^ indicated that the CO_2_ enrichment significantly delayed the larval stage but had no significant effects on the consumption of *H*. *armigera* individuals reared on wheat. Therefore, the response of *H*. *armigera* to elevated CO_2_ remains to be further elucidated. *H*. *armigera* exhibits CO_2_-sensitive receptor neurons in its labial palp that are temperature-compensated^30, 31^. Akbar *et al*.^16^ found that CO_2_ enrichment and increased temperature increased the consumption and metabolism of *H*. *armigera* fed an artificial diet by increasing protease activity and carbohydrates in the midgut. They suggested that *H*. *armigera* may cause more damage due to higher consumption rates under increased CO_2_.

Herbivores may exhibit different direct responses to elevated CO_2_. Elevated CO_2_ has no direct effects on the buckeye butterfly^32^. However, in the Asian corn borer fed an artificial diet, fitness-related parameters, including the larval duration, survivorship and pupal weight, were found to be adversely effected by CO_2_ enrichment, whereas food consumption was increased^20^. Wu *et al*.^14^ indicated that the fitness of *H*. *armigera* would be decreased by the a CO_2_-enriched atmosphere due to a delayed larval duration, increased individual fecundity, and increased consumption, as observed under elevated CO_2_ in *H*. *armigera* fed an artificial diet, and that the resultant damage would be serious. Akbar *et al*.^16^ suggested that increased CO_2_ could adversely affected the larval survival, larval duration and larval weight of *H*. *armigera* fed an artificial diet, whereas pupal weight and individual fecundity would be increased. In contrast, in experiments performed by Yin *et al*.^15^, elevated CO_2_ increased the larval period, fecundity and consumption of *H*. *armigera* fed an artificial diet. However, these differences compared with ambient CO_2_ conditions were not significant and suggested that the direct effect of elevated CO_2_ on *H*. *armigera* was small. In this previous study, the larval duration (10.7d), pupal duration (9.5d), mortality, and adult longevity of *H*. *armigera* fed an artificial diet under elevated CO_2_ levels showed trends similar to our results (Table 1), although the variations among the treatments were different. We believe that this difference may be due to two factors. First, all of our parameters were calculated using the age-stage, two-sex life table procedures. Different results are expected when naturally occurring variations resulting from precisely defining the stage structure and using data from both sexes are incorporated into the life table. Variations in behavior, physiology and susceptibility to stimuli that would otherwise would be omitted are included in the life table data. This aspect was not examined in the study by Yin *et al*.^15^. The second factor is the bootstrap techniques that are incorporated into the age-stage, two-sex life that are employed to estimate the means and standard errors of population parameters. The differences between the three CO_2_ concentrations were analyzed using paired bootstrap tests in the present study, whereas Yin *et al*.^15^ employ the least significant difference (LSD) using SPSS to obtain the means and SEs. The bootstrap techniques are based on the resampling procedure of deleting or repeatedly choosing all data for specific individuals^33^, moreover, as the sample size increases, the means of the samples will approach a normal distribution and will reduce the bias in population parameters^34^. Akca *et al*.^35^ demonstrated a difference between using general statistics and a bootstrap procedure to analyze the longevity and fecundity in a population. They showed that although the SEs calculated using general statistics are similar to those calculated with the bootstrap procedure, large differences in variances are essential in analysis of variance and significance tests. They then suggested that the bootstrap procedure should be used to analyze the representative variances and SEs of the population means. However, the bootstrap procedure has not been employed in all studies focusing on both the indirect and direct effects of elevated CO_2_ on *H*. *armigera*. Additionally, in the experiments of Yin *et al*.^29^ addressing the indirect effects of elevated CO_2_ on *H*. *armigera*, although the population parameters (*R*_0_, *T*, *r*) were analyzed based on an age-stage, two-sex life table, the jackknife technique was employed to estimate the means and standard errors of the population parameters. Efron^36^ noted that bootstrap techniques are more widely applicable and more dependable than the jackknife method and that they are better for estimating the variance of a sample median. Yu *et al*.^37^ reported that using the jackknife method would result in a zero net reproductive rate with the omission of a males, immature death, or nonreproductive females. Therefore, we recommend using bootstrap methods when estimating the population parameters of herbivorous insects. Additionally, the daily consumption rate was incorporated into the age-stage, two-sex life table in our study, which permits a precise description of potential consumption, and the sum of the stage-specific consumption of each instar was used to calculate to the larval consumption capacity. In previous studies, the effects of stage-specific mortality on the consumption of *H*. *armigera* have been ignored ^14–16, 27–29^. Here, we suggested that an age-stage, two-sex life table should be employed to analyze the fitness, including growth rate, fecundity and consumption, associated CO_2_ enrichment.

Our study is the first to correlate the direct effects of elevated CO_2_ on the life tables, consumption rates, population parameters and population projections of *H*. *armigera* using an age-stage, two-sex life table. The data were employed to explain differences between previous studies and our experiments when predicting the fitness of and population potential damage caused by *H*. *armigera* in response to elevated CO_2_ levels. The results of our experiments indicated that the increasing CO_2_ levels would adversely affect *H*. *armigera*. Additional studies on the long-term direct and indirect effects of elevated CO_2_ levels on *H*. *armigera* are still needed.

## Material and Methods

### Closed-dynamics CO_2_ chamber

All experiments were performed using a controlled environment the growth chamber (PRX-450D-30; Haishu Safe Apparatus, Ningbo, China). The apparatus was maintained at 27 ± 0.5 °C with 70 ± 5% RH, and a 14:10 (L:D) photoperiod, with 30,000 LX being provided by thirty-nine, 26 W fluorescent bulbs. The three tested atmospheric CO_2_ concentrations were 380 ppm, 550 ppm, and 750 ppm. A separate closed-dynamics chamber was used for each of the three CO_2_ levels. The growth chamber, which was equipped with in an automatic-control system to monitor and adjust the CO_2_ concentration every 20 min, is described in detail in Chen *et al*.^38^. The average CO_2_ concentration in each treatment was 380 ± 25, 550 ± 30, and 750 ± 38 ppm.

### *H*. *armigera* rearing procedure

The *H*. *armigera* colony was established from specimens originally collected in Wuhan City, Hubei Province, China, and was subsequently maintained by the Insect Ecology Laboratory of Huazhong Agricultural of University, Wuhan, Hubei Province, China. *H*. *armigera* larvae were fed a wheat germ and soybean powder-based artificial diet and allowed to develop in different chambers containing different levels of CO_2_.

One hundred and fifty newly oviposited eggs were used in each of the CO_2_ treatments. Ten eggs that were less than 2 days old were obtained from each of 15 randomly selected females. The newly hatched larvae were transferred to individual glass tubes (1 cm in diameter, 9 cm in height) containing specific amounts of the artificial diet and covered with 0.3-cm-diameter mesh netting to allow aeration and prevent the escape of larvae. The larvae were removed and transferred to tubes containing fresh diet at the beginning of the fifth instar and the beginning of the prepupal stages to avoid disturbance. Samples of the fresh artificial diet given to first- and fifth-instar larvae were weighed, dried and re-weighed to obtain baseline values of water content. The unconsumed artificial diets was weighted and dried at 80 °C for 72 h to measure water content. Data on the duration of larval instars, mortality and the consumption of each individual were recorded. All individuals were removed and weighted within 24 h of pupation and were then transferred to individual plastic cups (8 cm in diameter, 9 cm in height). Newly emerged adults were paired in plastic cups (8 cm in diameter, 9 cm in height) covered with mesh netting, which was used as an oviposition substrate, and supplied with a cotton wick saturated with a 30% honey solution as a nutrient source. The eggs were then counted and collected daily and transferred to a separate container. The newly emerged larvae were counted, removed, and placed in new rearing tubes. The number of viable eggs (hatched eggs) produced was equivalent to the number of emerged larvae, which was obtained by subtracting the emerged larvae from the total number of eggs. Only the viable eggs were included in the age-specific fecundity calculations. The survival rate, hatchability, and fecundity of each individual were recorded daily until the death of all individuals.

### Life table of *H*. *armigera*

All raw life history data were analyzed using age-stage, two-sex life table methods. The life history data were pooled and analyzed by the computer program TWOSEX-MSChart^39^. The age-stage specific survival rate (*s*_*xj*_) (where *x* = age and *j* = stage), the age-specific survival rate (*l*_*x*_), the age-stage fecundity (*f*_*xj*_), the age-specific fecundity (*m*_*x*_), and population parameters, including the intrinsic rate of increase (*r*), the net reproductive rate (*R*_0_), the finite rate of increase (*λ*), and the mean generation time (*T*) were calculated. According to the age-stage, two-sex life table theory, the age-specific survival rate (*l*_*x*_) is calculated as follows:

1 $${l}_{x}=\sum _{j=1}^{\beta }{s}_{xj}$$

(where *β* = the number of stages)^40^. The age-specific fecundity (*m*_*x*_) is calculated as follows:

2 $${m}_{x}=\frac{{\sum }_{j=1}^{\beta }{s}_{xj}{f}_{xj}}{{\sum }_{j=1}^{\beta }{s}_{xj}}$$

The net reproductive rate (*R*_0_) is calculated as follows:

3 $${R}_{0}=\sum _{x=0}^{\infty }{l}_{x}{m}_{x}$$

The intrinsic rate of increase (*r*) is calculated using the Euler-Lotka formula with the age indexed from day 0^41^:

4 $$\sum _{x=0}^{\infty }{e}^{-r(x+1)}{l}_{x}{m}_{x}=1$$

The finite rate (λ) is calculated as follows:

5 $${\rm{\lambda }}={e}^{r}$$

The mean generation time (*T*) is defined as the length time that is needed by a population to increase to *R*_0_-fold its current size when the stable rate of increase is reached, which is calculated as follows:

6 $$T=\frac{In{R}_{0}}{r}$$

The age-stage life expectancy (*e*_*xj*_), defined as the length time that an individual of age *x* and stage *j* is expected to survive, was estimated according to Chi and Su^42^. APOP is defined as the pre-oviposition period based on the female adult stage, whereas TPOP is considered the total time from birth to the initial oviposition. The standard errors of the development time, reproduction time, and population parameters were analyzed via a bootstrap approach with a sample size of 100,000^34, 43, 44^. The differences among different treatments were analyzed with a paired bootstrap test at the 5% significance level. All graphs were created using Sigma plot v. 12.0 software.

### Consumption rate of *H*. *armigera*

Individual consumption rates for each larva were recorded throughout their developmental period, and the data were incorporated into the age-stage, two-sex life table. Age-stage-specific consumption (*c*_*xj*_), which is defined as the daily consumption of all individuals of age *x* and stage *j*, integrates stage differentiation and variable consumption rates among individuals into the life table^45^. The age-specific consumption rate (*k*_*x*_) is defined as the mean amount of diet consumed by each *H*. *armigera* larvae at age *x* and is calculated as follows:

7 $${k}_{x}=\frac{{\sum }_{j=1}^{\beta }{s}_{xj}{c}_{xj}}{{\sum }_{j=1}^{\beta }{s}_{xj}}$$

where *β* is the number of life stages. The age-specific net consumption rate (*q*_*x*_) is the weighted consumption of *H*. *armigera* at age *x*; this parameter incorporates the survival rate and is calculated as follows:

8 $${q}_{x}={l}_{x}{k}_{x}$$

The net consumption rate (*C*_0_), which is the total consumption by an average individual during its life span, is calculated as follows:

9 $${c}_{0}=\sum _{x=0}^{\delta }{k}_{x}{l}_{x}$$

where δ is the last stage of the population. The transformation rate (*Q*_*p*_) is defined as the amount of diet needed by an *H*. *armigera* larva to produce a single newborn. Chi and Yang^46^ defined *Q*_*p*_ as $\,{Q}_{p}=\frac{{C}_{0}}{{R}_{0}}$, and stated that this is a demographic parameter that represents the consumption capacity of a pest population, including both sexes and individuals who died before reaching the adult stage. All consumption data were analyzed using the computer program CONSUME-MSChart^47^. The standard errors of the consumption values were also estimated using bootstrap techniques.

### Population and consumption projection of *H*. *armigera*

The program TIMING-MSChart^48^ was employed to simulate population growth and the consumption capacity over a period of 60 days. The data file used for projection was based on the two-sex life stable and age-stage-specific consumption (*c*_*xj*_). The age-stage, two-sex life table is capable of describing stage differentiation, and linking the *c*_*xj*_ with a life table is critical for understanding the potential consumption capacity. The initial population size was 10 individuals for all treatments. The consumption capacity at time *t* is calculated as follows:

10 $$p\,(t)=\sum _{j=1}^{\delta }(\sum _{x=0}^{\infty }{c}_{xj}{n}_{xj}(t))$$

where *c*_*xj*_ is the mean daily consumption rate of individuals at age *x* and stage *j*; and *n*_*xj*_ (*t*) is the number of individuals at age *x* and stage *j* at time *t*.

## References

[1] Fitt GP (1989). The ecology of Heliothis species in relation to agroecosystems. Annu. Rev. Entomol..

[2] Reigada C, Guimarães KF, Parra JRP (2016). Relative fitness of *Helicoverpa armigera* (Lepidoptera: Noctuidae) on seven host plants: a perspective for IPM in Brazil. J. Insect sci..

[3] Cunningham JP, Zalucki MP, West SA (1999). Learning in *Helicoverpa armigera* (Lepidoptera: Noctuidae): a new look at the behavior and control of a polyphagous pest. Bull Entomol. Res..

[4] IPCC. Climate change 2007: The Physical Science Basis Summary for Policymakers. IPCC WGI Fourth Assessment Report, 1–21 (2007).

[5] Liu JX (2015). CO_2_ enrichment and N addition increase nutrient loss from decomposing leaf litter in subtropical model forest ecosystems. Sci. Rep..

[6] Whittaker JB (1999). Impacts and responses at population level of herbivorous insects to elevated CO_2_. Eur. J. Entomol..

[7] Sun, Y. C., Guo, H. J. & Ge, F. Plant-aphid interactions under elevated CO_2_: some cues from aphid feeding behavior. *Front*. *Plant Sci*. **7**, 10.3389/fpls.2016.00502 (2016). 10.3389/fpls.2016.00502 PMC4829579 27148325

[8] Stiling P, Cornelissen T (2007). How does elevated carbon dioxide (CO_2_) affect plant-herbivore interactions? A field experiment and meta-analysis of CO_2_-mediated changes on plant chemistry and herbivore performance. Global Change Biol..

[9] Zavala JA, Nabity PD, Evan HD (2013). An emerging understanding of mechanisms governing insect herbivory under elevated CO_2_. Annu. Rev. Entomol..

[10] Landosky JM, Karowe D (2014). Will chemical defenses become more effective against specialist herbivores under elevated CO_2_?. Global Change Biol..

[11] Tuan SJ, Yeh CC, Atlihan R, Chi H (2016). Linking life table and predation rate for biological control: A comparative study of *Eocanthecona furcellata* (Hemiptera: Pentatomidae) fed on *spodoptera litura* (Lepidoptera: Noctuidae) and *Plutella xylostella* (Lepidoptera: Plutellidae). J. Econ. Entomol..

[12] Chi H (1988). Life-table analysis incorporating both sexes and variable development rates among individuals. Environ. Entomol..

[13] Chi H, Liu H (1985). Two new methods for the study of insect population ecology. Bull. Inst. Zool..

[14] Wu, G., Chen, F. J. & Ge, F. Direct effects of elevated cotton bollworm *Helicovrpa armigera* Hübner. *Acto*. *Ecol*. *Sin*. **26**, 1732–1738 (in Chinese) (2006).

[15] Yin J, Sun YC, Wu G, Ge F (2010). Effects of elevated CO_2_ associated with maize on multiple generations of the cotton bollworm, *Helicoverpa armigera*. Entomol. Exp. App..

[16] Akbar SMD, Pavani T, Nagaraja T, Sharma HC (2016). Influence of CO_2_ and temperature on metabolism and development of *Helicoverpa armigera* (Noctuidae: Leepidoptera). Environ. Entomol..

[17] Brooks GL, Whitteker JB (1998). Responses of multiple generations of *Gastrophysa viridula*, feeding on *Rumex obtusifolius*, to elevated CO_2_. Global Change Biol..

[18] Sun YC, Chen FJ, Ge F (2009). Elevated CO_2_ changes interspecific competition among three species of wheat aphids: Sitobion avenae, Rhopalosiphum padi, and Schizaphis graminum. Environ. Entomol..

[19] Stacey DA, Fellowes ME (2002). Influence of elevated CO_2_ on interspecific interactions at higher trophic levels. Global Change Biol..

[20] Xie H, Zhao L, Yang Q, Wang Z, He K (2015). Direct effects of elevated CO_2_ levels on the fitness performance of Asian corn Borer (Lepidoptera: Crambidae) for multigenerations. Environ. Entomol..

[21] Bezemer TM, Jones TH (1998). Plant-insect herbivore interactions in elevated atmospheric CO_2_ quantitative analyses and guild effects. Oikos.

[22] Chen FJ, Ge F, Parajulee MN (2005). Impact of elevated CO_2_ on tri-trophic interaction of *Gossypium hirsutum*, *Aphis gossypii*, and *Leis axyridis*. Environ. Entomol..

[23] Fajer ED, Bowers MD, Bazzaz FA (1989). The effects of enriched carbon dioxide atmosphere on plant-insect herbivore interactions. Science.

[24] Wang CZ, Dong JF (2001). Interspecific hybridization of *Helicoverpa armigera* and *H*.*assulta* (Lepidoptera:Noctuidae). Chinese Sci. Bull.

[25] Chi H, Mou DF, Lee CC, Smith CL (2016). Comments on the paper “Invariance of demographic parameters using total or viable eggs”. J. Appl. Entomol..

[26] Chi, H. *et al*. Finite predation rate: a novel parameter for the quantitative measurement of predation potential of predator at population level. Nature Precedings: hdl:10101/npre.2011.6651.1: posted 27 (2011).

[27] Chen FJ, Wu G, Ge F, Parajulee MN, Shrestha RB (2005). Effects of elevated CO_2_ and transgenic Bt cotton on plant chemistry, performance, and feeding of an insect herbivore, the cotton bollworm. Entomol. Exp. Appl..

[28] Wu G, Chen FJ, Ge F (2006). Response of multiple generations of cotton bollworm *Helicoverpa armigera* Hubner, feeding on spring wheat, to elevated CO_2_. J. Appl. Entomol..

[29] Yin J, Sun YC, Wu G, Parajulee M, Ge F (2009). No effects of elevated CO_2_ on the population relationship between cotton bollworm, *Helicoverpa armigera* Hübner (Lepidoptera: Noctuidae), and its parasitoid, *Microplitis mediator* Haliday (Hymenoptera: Braconidae). Agr. Ecosyst. Environ..

[30] Bonger F, Boppré M, Ernst KD, Boeckh J (1986). CO_2_ sensitive receptors on labial palps of *Rhodogastria* moths (Lepidoptera: Arctiidae): physiology, fine structure and central projection. J. Comp. Physioy. A..

[31] Stange G, Wong C (1993). Moth response to climate. Nature.

[32] Fajer ED, Bowers MD, Bazzaz FA (1991). The effects of enriched CO_2_ atmospheres on the buckeye butterfly. Junonia coenia. Ecology.

[33] Huang YB, Chi H (2012). Assessing the application of the jackknife and bootstrap techniques to the estimation of the variability of the net reproductive rate and gross reproductive rate: a case study in *Bactrocera cucurbitae* (Coquillett) (Diptera: Tephritidae). Turk. J. Agric. For..

[34] Polat A, Atlihan R, Okut H, Chi H (2015). Demographic assessment of plant cultivar resistance to insect pests: a case study of the dusky-veined walnut aphid (Hemiptera: Callaphididae) on five walnut cultivars. J. Econ. Entomol..

[35] Akca I, Ayvaz T, Yazici E, Smith CL, Chi H (2015). Demography and population projection of *Aphis fabae* (Hemiptera: Aphididae): with additional comments on life table research criteria. J. Econ. Entomol..

[36] Efron BB (1979). Bootstrap methods: another look at the jackknife. Ann. Stat..

[37] Yu LY (2013). Demographic analysis, a comparison of the jackknife and bootstrap methods, and predation projection: a case study of *Chrysopa pallens* (Neuroptera: Chrysopidae). J. Econ. Entomol..

[38] Chen, F. J., Ge, F. & Su, J. W. An improved open-top chamber for research on the effects of elevated CO_2_ on agricultural pests in field. *Chin*. *J*. *Ecol*. **24**, 585–590 (in Chinese) (2005).

[39] Chi, H. Core Team. TWOSEX-MSChart: A Computer Program for the Age-stage, Two-sex Life Table Analysis. National Chung Hsing University, Taichung, Taiwan. URL http://140.120.197.173/Ecology/ (2017).

[40] Tuan SJ (2016). Survival and reproductive strategies in two-spotted spider mites: demographic analysis of arrhenotokous parthenogenesis of *Tetranychus urticae* (Acari: Tetranychidae). J. Econ. Entomo..

[41] Goodman D (1982). Optimal life histories, optimal notation, and the value of reproductive value. Am. Nat..

[42] Chi H, Su HY (2006). Age-stage, two-sex life tables of *Aphidius gifuensis* (Ashmead) (Hymenoptera: Braconidae) and its host *Myzus persicae* (Sulzer) (Homoptera: Aphididae) with mathematical proof of the relationship between female fecundity and the net reproductive rate. Environ. Entomol..

[43] Efron, B. & Tibshirani, R. J. An Introduction to the Bootstrap. Chapman & Hall, New York (1993).

[44] Johnson WR (2001). An introduction to bootstrap. Teach. Stat..

[45] Jha, R. K., Tuan, S. J., Chi, H. & Tang, L. C. Life table and consumption capacity of corn earworm, *Helicoverpa armigera*, fed asparagus, *Asparagus officinalis*. *J*. *Insect Sci*. **14**, Available online: http://www.insecscience.org/14.34 (2014). 10.1093/jis/14.1.34 PMC4206235 25373181

[46] Chi H, Yang TC (2003). Two-sex life table and predation rate of *Propylaea japonica* Thunberg (Coleoptera: Coccinellidae) fed on *Myzus persicae* (Sulzer) (Homoptera: Aphididae). Environ. Entomol..

[47] Chi, H. Core Team. CONSUME-MSChart: A Computer Program for the Age-stage, Two-sex Life Table Analysis. National Chung Hsing University, Taichung, Taiwan. URL http://140.120.197.173/Ecology/Download/CONSUME-MSChart.rar (2017).

[48] Chi, H. Core Team. Timing-MSChart: A Computer Program for the Population Projection Based on Age-stage, Two-sex Life Table. National Chung Hsing University, Taichung, Taiwan. URL http://140.120.197.173/Ecology/Download/TIMING-MSChart.rar (2017).

